# On the accurate computation of expected modularity in probabilistic networks

**DOI:** 10.1038/s41598-025-99114-5

**Published:** 2025-05-30

**Authors:** Xin Shen, Matteo Magnani, Christian Rohner, Fiona Skerman

**Affiliations:** 1 InfoLab, Department of Information Technology, Uppsala University, 75105 Uppsala, Sweden; 2 Department of Mathematics, Uppsala University, 75105 Uppsala, Sweden

**Keywords:** Modularity calculation, Probabilistic networks, Algorithms, Applied mathematics, Computational science, Mathematics and computing, Computer science

## Abstract

Modularity is one of the most widely used measures for evaluating communities in networks. In probabilistic networks, where the existence of edges is uncertain and uncertainty is represented by probabilities, the expected value of modularity can be used instead. However, efficiently computing expected modularity is challenging. To address this challenge, we propose a novel and efficient technique ($\textrm{FPWP}$) for computing the probability distribution of modularity and its expected value. In this paper, we implement and compare our method and various general approaches for expected modularity computation in probabilistic networks. These include: (1) translating probabilistic networks into deterministic ones by removing low-probability edges or treating probabilities as weights, (2) using Monte Carlo sampling to approximate expected modularity, and (3) brute-force computation. We evaluate the accuracy and time efficiency of $\textrm{FPWP}$ through comprehensive experiments on both real-world and synthetic networks with diverse characteristics. Our results demonstrate that removing low-probability edges or treating probabilities as weights produces inaccurate results, while the convergence of the sampling method varies with the parameters of the network. Brute-force computation, though accurate, is prohibitively slow. In contrast, our method is much faster than brute-force computation, but guarantees an accurate result.

## Introduction

Uncertainty is an inherent property when modelling a system as a network because of randomness of the system, inaccuracy of measurements, or their interpretation. System randomness can be found, for example, in computer networks where network links can be unreliable. Inaccuracy of measurements is ubiquitous, for example when estimating interaction probabilities in protein networks^1–3^ or assessing the existence of social relations in social networks^4,5^. Uncertainty is modelled associating each edge with a probability of existence, forming a *probabilistic network*^6–14^. When the existence of all edges is certain, we talk of *deterministic networks*^8,9,15–18^.

This paper focuses on the problem of calculating expected modularity in probabilistic networks. Modularity is a measure of the ratio of edges falling within partitions minus approximately the expected ratio in an equivalent network with edges placed at random^19^. Higher values of modularity often indicate that the input partitioning provides a good representation of the modules constituting the network. Therefore, despite some limitations of modularity as an objective function^20–23^, modularity optimization^24^ has emerged as one of the most popular approaches for the analysis of deterministic networks.

In probabilistic networks, modularity is not represented by a single value but by a distribution of modularity values arising from all combinations of edges in the network. These combinations are called *possible worlds*, each having a different modularity and probability in general. There are no studies proposing specific algorithms to compute the expected modularity, which means that we currently have to rely on general methods. One way of computing expected modularity in probabilistic networks is to calculate it over all possible worlds^8,9,15,25^, and calculate its expected value. This approach is accurate; in fact, it is the only known approach producing the correct value of expected modularity, modulo numerical approximations. However, this approach is also computationally impractical as it involves calculating modularity $2^m$ times, where *m* is the number of edges in the network. Another approach is to sample $\theta$ possible worlds, then calculate modularity for each sample and take the average value^3^. This approach can be very fast, but prioritizes execution time over accuracy: one can speed up the execution by choosing a lower $\theta$, but at the cost of obtaining a potentially inaccurate result without a guaranteed approximation error. Other general ways of handling probabilistic networks, which can also be applied to modularity computation, include regarding edge probabilities as edge weights or setting a threshold to convert probabilistic networks to deterministic networks by removing edges with probability lower than the threshold. However, we show that these approaches are not appropriate for this specific task. Regarding probabilities as weights generally leads to wrong results, as we show in our experiments. Setting a suitable threshold is also a complicated problem as it requires prior knowledge of the network structure and the chosen threshold greatly affects the resulting modularity^16^. In fact, in this paper we experimentally evaluate all the aforementioned approaches, and show that there is no known simple and general way of computing the expected value of modularity other than enumerating and computing modularity in all possible worlds, without a risk of obtaining inaccurate results.

Because of the limitations of existing approaches, in this paper we also introduce a new method for expected modularity computation. The novelty of this work is a new approach to exactly compute the modularity distribution without enumerating all possible worlds. Our method consists in partitioning the possible worlds so that for each partition we can (1) easily compute the expected value of modularity for the possible worlds in that partition, and (2) quickly compute the probability of the partition. The intuition behind our method is that, given a network and a clustering of its nodes, the value of modularity only depends on the number of edges inside and across communities, and not on the specific nodes incident to each edge. Therefore, we can group all possible worlds with the same numbers of in- and across-community edges into the same partition, obtaining that all possible worlds in each partition will have the same value of modularity. This would then also be the same as the expected modularity for the partition. This approach allows us to compute the correct value of expected modularity without having to compute modularity in all $2^m$ possible worlds, and to apply methods for the fast computation of probabilities from the Poisson Binomial distribution to the problem of expected modularity computation. Our method reduces time complexity from exponential to polynomial compared to the brute-force approach. Different from e.g. sampling, whose accuracy depends on the number of samples, our method always returns an accurate result, and its execution time does not depend on the edge probability distribution. Thanks to its ability to return an accurate result in polynomial time, our method also allows us to evaluate the traditional approaches used to analyse probabilistic networks but not providing guarantees on their accuracy, that is, sampling, thresholding, and weighting, when used to estimate expected modularity.

The rest of the paper is organized as follows: after reviewing the background we present all the evaluated approaches, including the new algorithm introduced in this work. Then we present a thorough simulation-based experimental evaluation on random and real-world networks with different properties. We conclude with a summary and discussion of our results. The main notation used in the paper is summarized in Table 1.

Please note that while this work is motivated by the importance of community detection, here we focus on the foundational problem of computing expected modularity given an input clustering (that is, modularity *computation*) and not on how to use modularity to identify good clusterings (that is, modularity *optimization*). In this paper a clustering is always given as an input of the algorithm, and expected modularity is computed for the input clustering. We also note that probabilistic networks and random graph models are related but distinct concepts. A probabilistic network represents a specific real-world system, and given two nodes in that network, an edge between them either exists or not in the real world. Probabilities represent our ignorance with respect to the state of the real world.

**Table 1:** Summary of notations.

Probabilistic network	$\mathscr {G}$
Deterministic network	*G*
Number of nodes in $\mathscr {G}$	*n*
Number of edges in $\mathscr {G}$	*m*
Set of nodes in $\mathscr {G}$	*V*
Set of edges in $\mathscr {G}$	*E*
Edge between node *i* and node *j* in $\mathscr {G}$	$e_{ij}$
Probability of edge $e_{ij}$	$p_{ij}$
Edge between node *i* and node *j* in *G*	$l_{ij}$
Set of possible worlds in $\mathscr {G}$	$W =\{w_1,w_2,\ldots ,w_{2^m}\}$
Probability of possible world *w*	*Pr*(*w*)
Set of communities in $\mathscr {G}$	$\mathscr {C}=\{c_1,c_2,\ldots ,c_k\}$
Number of communities in $\mathscr {C}$	*k*
Set of nodes in community *c*	$V_{c}$
Set of edge probabilities in $\mathscr {G}$	$P=\{p_1,p_2,\ldots ,p_{2^m}\}$
Set of partitions of possible words in $\mathscr {G}$	$D=\{d_1,d_2,\ldots ,d_s\}$

## Background

### Probabilistic networks

Consider a probabilistic network $\mathscr {G}=(V,E,p)$, where *V* corresponds to the set of nodes in $\mathscr {G}$, *E* represents the set of edges, and $p: E \rightarrow (0,1]$ is a function that assigns probabilities to edges. We use $e_{ij}$ to indicate the edge between nodes *i* and *j*, $p_{ij}$ as a shorthand for $p({e_{ij}})$, and we notate $|E|=m$.

*Possible worlds semantics* interprets a probabilistic network as a generative model for deterministic networks, called possible worlds, each of which associated with its probability of being observed^16,25,26^. That is, we represent $\mathscr {G}$ with the set $\{G=(V,E_G)\}_{E_G \subseteq E}$ of all possible deterministic networks in $\mathscr {G}$ with their associated probabilities. As the edge probabilities are considered to be independent of each other^27,28^, the probability of observing such a set is:

1 $$\begin{aligned} \begin{aligned} Pr(G)=\prod _{e\in E_G}p(e)\prod _{e\in E \backslash E_G}(1-p(e)) \ . \end{aligned} \end{aligned}$$

There are $2^{m}$ distinct networks in $\mathscr {G}$.

#### Modularity

A common way to identify communities in deterministic networks is to maximize the modularity score, which rewards solutions capturing many of the edges within communities. Giving a community labeling of vertices $x$, the modularity for an undirected network is given by:

2 $$\begin{aligned} \begin{aligned} Q=\frac{1}{2M}\sum _i \sum _j\left( A_{ij}-\frac{k_ik_j}{2M}\right) \delta (x_i,x_j) \ , \end{aligned} \end{aligned}$$

where *M* is the number of edges, *A* is the adjacency matrix, $k_i$ represents the degree of node *i*, $x_i$ is the label of node *i*, and $\delta (x_i,x_j)$ is the Kronecker delta function, which equals 1 when its arguments are the same and 0 otherwise. Modularity is a function of a network and a community assignment (that is, a partitioning of its nodes). However, it is common not to show its parameters and only write the function name (*Q*), to simplify the notation.

Modularity can also be expressed in the following form^24^, which is the one used in our method:

3 $$\begin{aligned} \begin{aligned} Q=\sum _{c \in \mathscr {C}}\frac{|e_c|}{(|e_c|+|e_{c,\bar{c}}|+|e_{\bar{c}}|)}-\left( \frac{2|e_c|+|e_{c,\bar{c}}|}{2(|e_c|+|e_{c,\bar{c}}|+|e_{\bar{c}}|)}\right) ^2 \ , \end{aligned} \end{aligned}$$

where ${\mathscr {C}}$ is the set of communities, $V_{c}$ is the set of nodes in community $c \in \mathscr {C}$, and

4 $$\begin{aligned} \begin{aligned} e_c&=\{e_{ij}\ |\ i,j \in V_{c}\},\\ e_{c,\bar{c}}&=\{e_{ij}\ |\ (i \in V_{c} \wedge j \notin V_{c}) \vee (j \in V_{c} \wedge i \notin V_{c})\}, \\ e_{\bar{c}}&=\{e_{ij}\ |\ i,j \notin V_{c}\}. \end{aligned} \end{aligned}$$

**Fig. 1:**
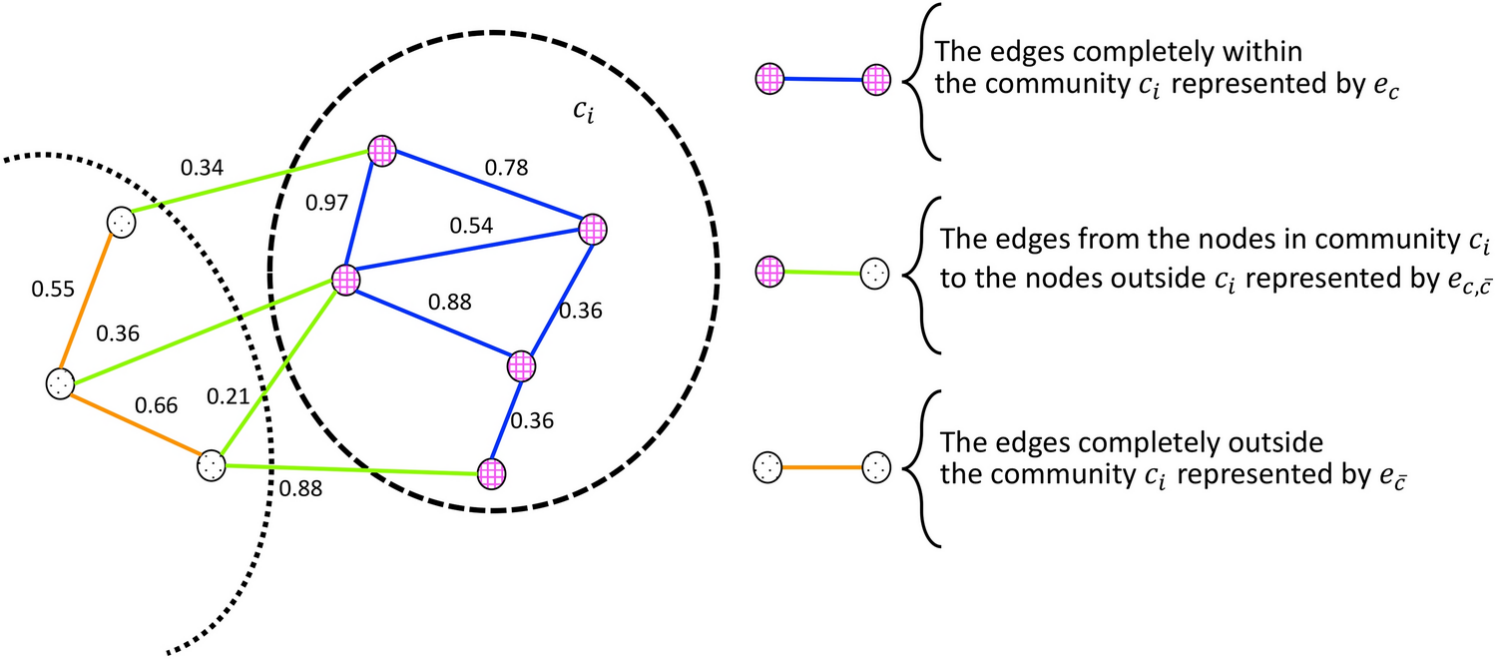
Edge partitions used in the community-based definition of modularity, for a community $c_i$.

An example of $e_c$, $e_{c,\bar{c}}$, and $e_{\bar{c}}$ for a community $c_i$ is presented in Fig. 1.

#### Expected modularity

A deterministic network $G_w$ in $\mathscr {G}$ can be considered as one possible world. From Eq. (1), we know the probability of each possible world in $\mathscr {G}$ ]. Let ${\textbf {Q}}$ be the discrete distribution of *Q* on the $2^m$ possible worlds. The expected value of ${\textbf {Q}}$ is:

5 $$\begin{aligned} \begin{aligned} E({\textbf {Q}})=\sum _{w=1}^{2^m} Q_w Pr(w), \end{aligned} \end{aligned}$$

where $Q_w$ is the modularity value of deterministic network $G_w$ (that is, possible world *w*).

#### Entropy ratio

The entropy^29,30^ $H(\mathscr {G})$ of a probabilistic network $\mathscr {G}$ is defined as the joint entropy of its edges *H*(*e*) for all $e \in E$. Here we use entropy to represent the different levels of uncertainty of a network. The larger entropy, the more uncertainty. Due to the edge independence, the formula of entropy is $H(\mathscr {G})=-\sum _{i<j}p_{ij}\log p_{ij}-\sum _{i<j}q_{ij}\log q_{ij}$, where $q_{ij}=1-p_{ij}$. To easily compare networks with different sizes, we normalize such entropy by dividing by the number of edges. We call this *entropy ratio*, where entropy ratio =$\frac{|\mathscr {H}(\mathscr {G})|}{m}$, and its range is [0,1].

## Computation methods

### Brute-force

The brute-force method to compute expected modularity directly uses Eq. (5), calculating the modularity on every possible world and their expected value^31^. Therefore, the time complexity of this method is at least exponential on the size of the network. While impractical, this method returns the correct value of expected modularity, and can thus be used on small networks to evaluate the accuracy of other approaches.

#### Sampling

The sampling method called Monte Carlo estimator^32^ first generates $\theta$ samples from the probability distribution over the possible worlds, then calculates modularity for each sample *t* (which we notate $Q_t$) and computes the average modularity value:

6 $$\begin{aligned} \begin{aligned} Q=\frac{1}{\theta }\sum _{t=1}^\theta Q_t \ . \end{aligned} \end{aligned}$$

Once the parameter $\theta$ has been specified, creating samples from the ensemble is straightforward. The detailed algorithm is depicted in Algorithm 1. Note that we do not use Naive Monte Carlo sampling, that is known to have a higher variance^33^.

**Algorithm 1:**
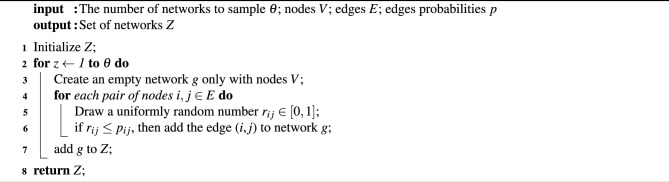
Sampling method

#### Thresholding

Thresholding is a simple and general method used in community detection in probabilistic networks. Its purpose is to transform probabilistic networks into deterministic networks, so that existing methods for deterministic networks can be applied. After setting a threshold, we remove edges whose probability is lower than the threshold and consider the others as deterministic.

### Possible-World Partitioning (PWP)

The Possible-World Partitioning for Expected Modularity ($\textrm{PWP}$) algorithm, that we introduce in this work, groups the possible worlds into partitions so that all possible worlds inside the same partition have the same value of modularity. For a given community assignment $\mathscr {C}$ and using the alternative definition of modularity in Eq. (3), expected modularity can be rewritten as:

7 $$\begin{aligned} \begin{aligned} E({\textbf {Q}})=\sum _{w=1}^{2^m} \sum _{c \in \mathscr {C}} Q_c^w Pr(w) \ , \end{aligned} \end{aligned}$$

where *w* indicates a possible world corresponding to deterministic network $G_w$, *Pr*(*w*) is the probability of this possible world,

$$\begin{aligned} Q_c^w = \frac{|e_c^w|}{(|e_c^w|+|e_{c,\bar{c}}^w|+|e_{\bar{c}}^w|)}-\left( \frac{2|e_c^w|+|e_{c,\bar{c}}^w|}{2(|e_c^w|+|e_{c,\bar{c}}^w|+|e_{\bar{c}}^w|)}\right) ^2 \ , \end{aligned}$$

and $e_c^w$, $e_{c,\bar{c}}^w$, and $e_{\bar{c}}^w$ refer to $e_c$, $e_{c,\bar{c}}$, and $e_{\bar{c}}$ in $G_w$.

After rearranging the two sums:

8 $$\begin{aligned} \begin{aligned} E({\textbf {Q}})&=\sum _{c \in \mathscr {C}} \sum _{w=1}^{2^m} Q_c^w Pr(w) \ , \end{aligned} \end{aligned}$$

we can partition the $2^m$ possible worlds in a way that allows us to process all the possible worlds in each partition without iterating over them. In particular, the key idea of our approach to reduce computational complexity is to define these partitions so that the possible worlds within the same partition have the same modularity. We do this by defining partitions whose possible worlds have the same number of edges in $e_c^w$, $e_{c,\bar{c}}^w$, and $e_{\bar{c}}^w$, respectively. Note that these sets are disjoint and in general have different sizes. We notate $d^{xyz}$ the partition containing all possible worlds *w* where $|e_c^w| = x$, $|e_{c,\bar{c}}^w| = y$, and $|e_{\bar{c}}^w| = z$. This partition contains $$\left( {\begin{array}{c}T_x\\ x\end{array}}\right) \cdot \left( {\begin{array}{c}T_y\\ y\end{array}}\right) \cdot \left( {\begin{array}{c}T_z\\ z\end{array}}\right)$$ possible worlds, where $T_x = |e_c|$, $T_y = |e_{c,\bar{c}}|$, and $T_z = |e_{\bar{c}}|$ are the (maximum) number of edges in the three parts, respectively. We can then write:

9 $$\begin{aligned} \begin{aligned} {E({\textbf {Q}})=\sum _{c \in \mathscr {C}} \sum _{x=0}^{T_x} \sum _{y=0}^{T_y} \sum _{z=0}^{T_z} Q_c^{xyz} Pr( d^{xyz}}) \ , \end{aligned} \end{aligned}$$

where $Q_c^{xyz}$ is the constant value of $Q_c^w$ in all possible worlds $w \in d^{xyz}$, that is:

10 $$\begin{aligned} \begin{aligned} Q_c^{xyz} = \frac{x}{(x+y+z)}-\left( \frac{2x+y}{2(x+y+z)}\right) ^2 . \end{aligned} \end{aligned}$$

Notice that computing expected modularity using Eq. (9) we only need to iterate over $|\mathscr {C}|T_xT_yT_z$ terms instead of $2^m$, and for each term the value of $Q_c^{xyz}$ can be computed in constant time. The probability of partition $d^{xyz}$ can be expressed as a product of probabilities, because $e_c^w$, $e_{c,\bar{c}}^w$, and $e_{\bar{c}}^w$ are always disjoint sets:

11 $$\begin{aligned} \begin{aligned} {Pr(d^{xyz}) =Pr(|e_c^w| = x \wedge |e_{c,\bar{c}}^w| = y \wedge |e_{\bar{c}}^w| = z) = Pr(|e_c^w|=x) \cdot Pr(|e_{c,\bar{c}}^w|=y) \cdot Pr(|e_{\bar{c}}^w|=z) \ .} \end{aligned} \end{aligned}$$

These probabilities can be computed using the definition of Poisson Binomial distribution. In the following equations, *A* is an element of $F_x$, $F_y$ or $F_z$, where $F_{x}$ is the set of all subsets of $e_c$ of size *x*, $F_{y}$ is the set of all subsets of $e_{c,\bar{c}}$ of size *y*, $F_{z}$ is the set of all subsets of $e_{\bar{c}}$ of size *z*.

12 $$\begin{aligned} \begin{aligned} Pr(|e_c^w|=x)= \sum _{A \in F_x }\prod _{e\in A}p(e)\prod _{e\in e_c\backslash A}(1-p(e)) \ , \end{aligned} \end{aligned}$$

13 $$\begin{aligned} \begin{aligned} Pr(|e_{c,\bar{c}}^w|=y)= \sum _{A \in F_y }\prod _{e\in A}p(e)\prod _{e\in e_{c,\bar{c}}\backslash A}(1-p(e)) \ , \end{aligned} \end{aligned}$$

14 $$\begin{aligned} \begin{aligned} Pr(|e^w_{\bar{c}}|=z)= \sum _{A \in F_z }\prod _{e\in A}p(e)\prod _{e\in e_{\bar{c}}\backslash A}(1-p(e)) \ . \end{aligned} \end{aligned}$$

**Fig. 2:**
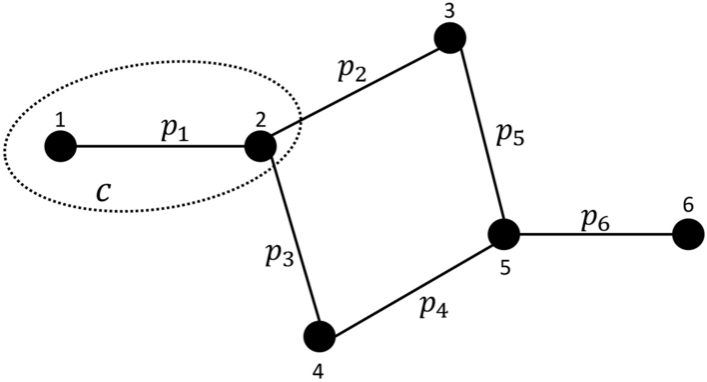
A probabilistic network with a community *c*.

**Fig. 3:**
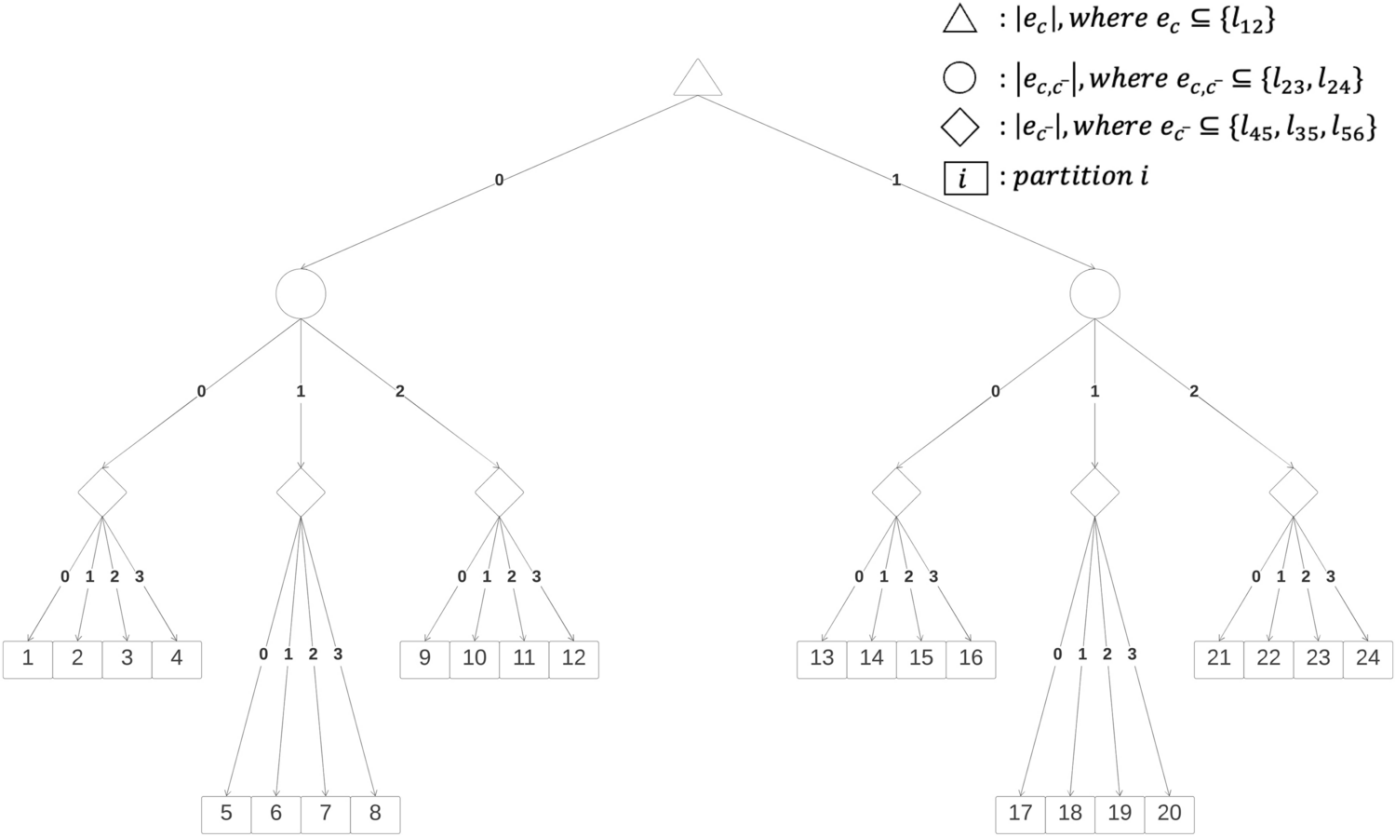
A tree enumerating all partitions of possible worlds defined by community *c*.

**Fig. 4:**
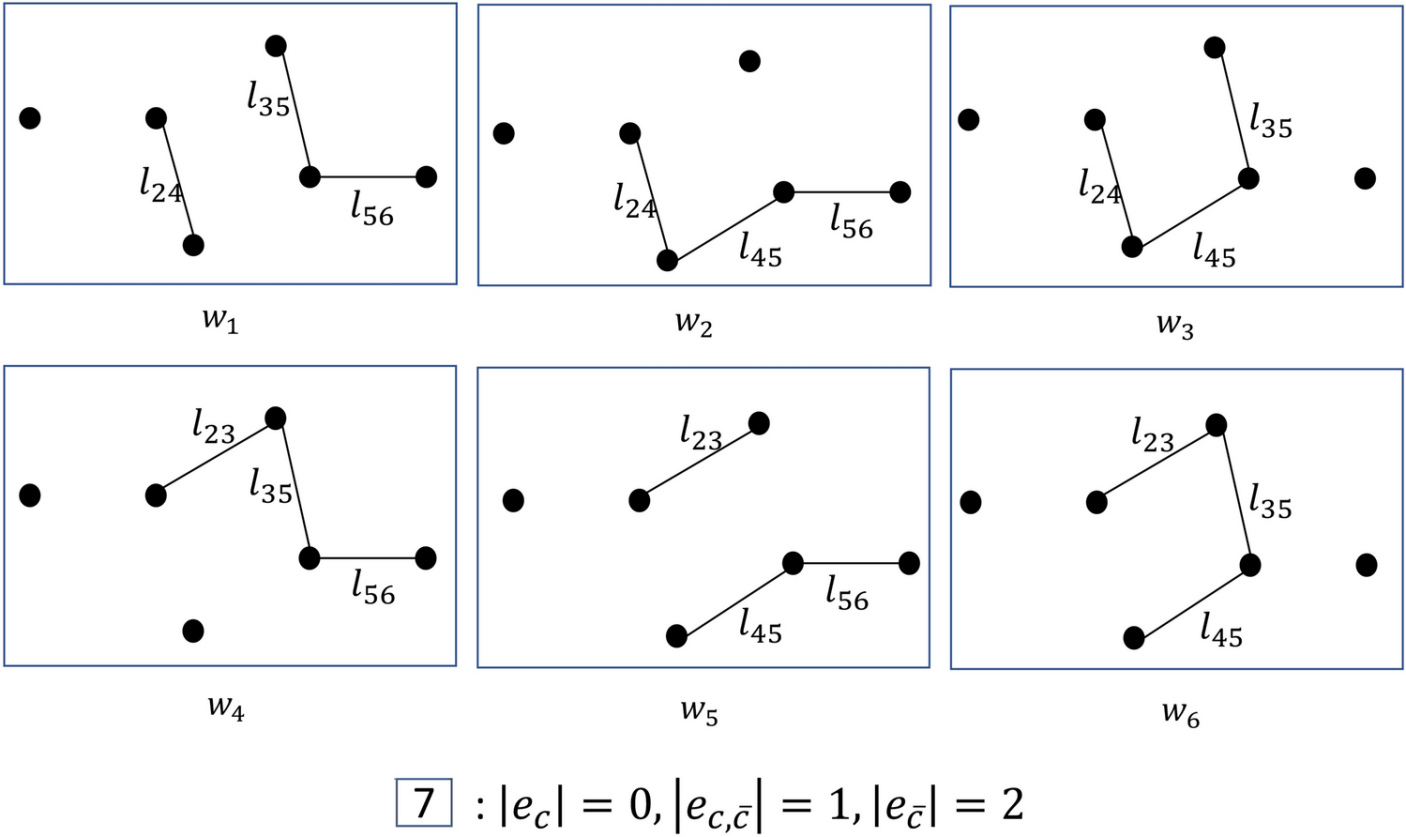
All possible worlds in partition 7. $|e_c^w|, |e_{c,\bar{c}}^w|$, and $|e_{\bar{c}}^w|$ are constant inside the partition.

As an example of how the partitions are defined, consider the probabilistic network in Fig. 2. Figure 3 shows a tree whose leaves enumerate all partitions $d^{xyz}$ defined by community *c*. The two branches from the top triangle represent respectively all the possible worlds where $e_c^w$ contains no edges ($x=0$) and all the possible worlds where $e_c^w$ contains one edge ($x=1$), in this case edge $l_{12}$. As we move down the tree, the circles in the second level represent all the possible worlds where $e_{c,\bar{c}}^w$ contains no edges ($y=0$), one edge ($y=1$, in particular $l_{23}$ or $l_{24}$), and two edges ($y=2$, in this case both $l_{23}$ and $l_{24}$). The same with the diamonds in the third level representing possible worlds with a specific number of edges in $e_{\bar{c}}^w$. The squares at the bottom of the figure represent all partitions. Figure 4 shows all possible worlds in partition 7, where $|e_c^w|=0, |e_{c,\bar{c}}^w|=1, |e_{\bar{c}}^w|=2$.

$\textrm{PWP}$ is detailed in Algorithm 2. In lines 6, 8 and 10, we use Eqs. (12), (13) and (14) to calculate $Pr(|e_c^w|=x)$, $Pr(|e_{c,\bar{c}}^w|=y)$ and $Pr(|e_{\bar{c}}^w|=z)$ separately.

While $\textrm{PWP}$ can already be considered a usable algorithm, because it reduces the number of modularity computations from exponential to polynomial, its worst-case time complexity is still $O(km2^m)$. We can break the analysis down into two parts. Lines 3 to 10 concern the exact calculation of probabilities for each partition. The time complexities of *Pr*(*x*), *Pr*(*y*), and *Pr*(*z*) are $O(2^m)$. From Eq. 9, we can see that *x* ranges from 1 to $T_x$, *y* ranges from 1 to $T_y$, and *z* ranges from 1 to $T_z$. $T_x$, $T_y$, and $T_z$ sum to *m*, so the overall complexity is $O(m2^m)$. Lines 11 to 15 involve looping over all partitions, which has a time complexity of $O(m^3)$. Notice that in the worst-case scenario, the number of edges in $e_c$, $e_{c,\bar{c}}$, and $e_{\bar{c}}$ are similar to each other. Here the number of edges in $e_c$, $e_{c,\bar{c}}$, and $e_{\bar{c}}$ have the same upper bound *m*. Both parts are inside the community loop on line 2, so the time complexity of the whole algorithm is $O(k(m2^{m} + m^3)) = O(km2^{m})$, where *k* is the number of communities. Notice that the time complexity solely depends on the number of communities and edges, and remains unaffected by the assignment of edge probabilities.

### Fast/Fourier Possible-World Partitioning (FPWP)

While PWP practically only can be used in small networks, its partitioning approach, combined with the Poisson Binomial distribution, enables a further reduction in computational complexity. The Poisson Binomial distribution can be expressed in closed-form using the Discrete Fourier Transform^34^;

15 $$\begin{aligned} \begin{aligned} {Pr(X=k)=\frac{1}{n+1}\sum _{l=0}^{n} C^{-lk}\prod _{i=1}^{n} (1+(C^l-1)p_{i}^k) \ }\end{aligned} \end{aligned}$$

where $$C=exp(j\frac{2\pi }{n+1})$$ and $$j=\sqrt{-1}$$.

This leads to a version of our method that we call Fast/Fourier Possible World Partitioning for Expected Modularity ($\textrm{FPWP}$), replacing Eqs. (12), (13), (14) by the following Eqs. (16), (17), (18), respectively:

16 $$\begin{aligned} \begin{aligned} Pr(|e_c^w|=x)&{\approx } {= }\frac{1}{T_{x}+1}\sum _{l=0}^{T_{x}} C^{-lx}\prod _{\alpha =1}^{T_{x}} (1+(C^l-1)p_{\alpha }^x) \ , \end{aligned} \end{aligned}$$

17 $$\begin{aligned} \begin{aligned} Pr(|e_{c,\bar{c}}^w|=y)&{\approx } {= }\frac{1}{T_{y}+1}\sum _{l=0}^{T_{y}} C^{-ly}\prod _{\alpha =1}^{T_{y}} (1+(C^l-1)p_{\alpha }^y) \ , \end{aligned} \end{aligned}$$

18 $$\begin{aligned} \begin{aligned} Pr(|e_{\bar{c}}|=z)&{\approx } {= }\frac{1}{T_{z}+1}\sum _{l=0}^{T_{z}} C^{-lz}\prod _{\alpha =1}^{T_{z}} (1+(C^l-1)p_{\alpha }^z) \ . \end{aligned} \end{aligned}$$

$\textrm{FPWP}$ is also detailed in Algorithm 2. In this case, differently from $\textrm{PWP}$, in lines 6, 8 and 10 we use Eqs. (16),  (17) and (18) to calculate $Pr(|e_c^w|=x)$, $Pr(|e_{c,\bar{c}}^w|=y)$ and $Pr(|e_{\bar{c}}^w|=z)$ separately.

**Algorithm 2:**
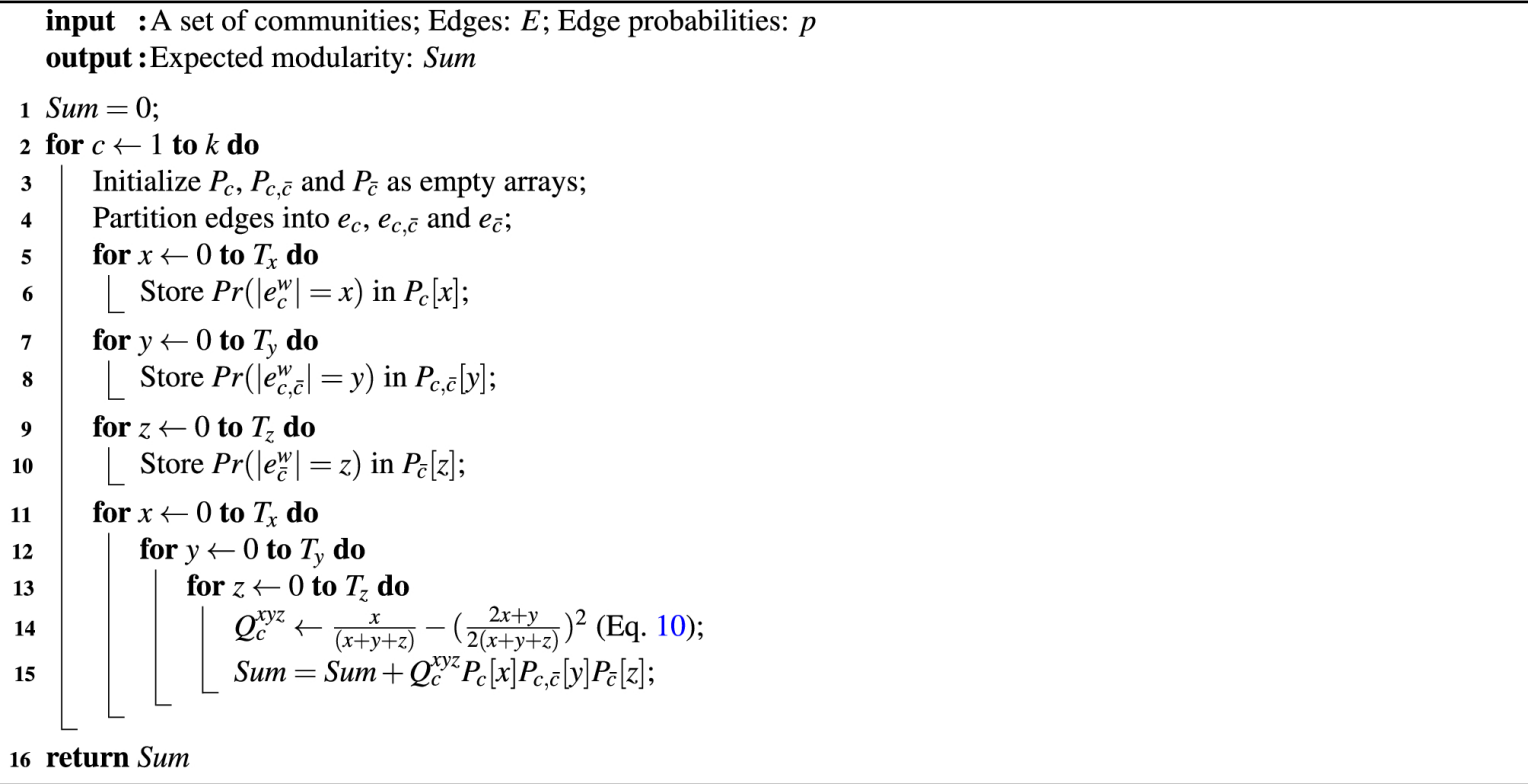
$$\mathrm {(F)PWP}$$

The worst-case time complexity of $\textrm{FPWP}$ is $O(km^3)$. We can break the analysis down into two main parts. Lines 3 to 10 concern the probabilities for each partition. The time complexities of *Pr*(*x*), *Pr*(*y*), and *Pr*(*z*) are $O(m^2)$. From Eq. (9), we can see that *x* ranges from 1 to $T_x$, *y* ranges from 1 to $T_y$, and *z* ranges from 1 to $T_z$. $T_x$, $T_y$, and $T_z$ sum to *m*, so the overall complexity is $O(m^3)$. Lines 11 to 15 involve looping over all partitions, which has a time complexity of $O(m^3)$. Notice that in the worst-case scenario, the number of edges in $e_c$, $e_{c,\bar{c}}$, and $e_{\bar{c}}$ are similar to each other. Here the number of edges in $e_c$, $e_{c,\bar{c}}$, and $e_{\bar{c}}$ have the same upper bound *m*.

Both parts are inside the community loop on line 2, so the time complexity of the whole algorithm is $O(km^3),$ where *k* is the number of communities. Notice that the time complexity solely depends on the number of communities and edges, and remains unaffected by the assignment of edge probabilities.

## Evaluation

The brute-force method produces the correct result and can thus be used to evaluate the other approaches. However, it can only be used on very small networks. Therefore, we start our evaluation showing that our method is as accurate as the brute-force method, but is several orders of magnitude faster. In this way, we can then use our method to evaluate the other possible ways to estimate expected modularity: weighting, thresholding, and sampling. We also study the behavior of our algorithm when we vary the number of communities, the distribution of community sizes, and the type of input network.

All the experiments except the comparison with the brute-force method have been performed on a macOS system, with max CPU frequency 2.4GHz. The comparison with the brute-force method has been performed on a Linux system, with max CPU frequency 5GHz. Both systems have the same 32GB memory capacity.

*Data.* In our experiments we use both synthetic datasets, to control and examine the properties of the data that may affect running time and accuracy of the tested algorithms, and real-world datasets.

To generate synthetic datasets, we use Stochastic Block Model (SBM), Forest Fire Network (FFN), Barabási-Abert (BA), Small World (SW), and Erdős-Rényi (ER). The parameters used to generate specific random networks are specified later in this section for each experiment.

As real datasets, we use the Enron email network, an Online Social Network (OSN), a Protein-Protein Interaction (PPI) network, and a Collaboration network, summarized in Table 2. The data sources used to generate these networks have been commonly used in the literature on probabilistic networks. In particular, two datasets (Enron, PPI) have been directly taken from the literature, while for those not provided by the authors we obtained probabilistic networks from the same sources following similar procedures and reproducing similar network statistics. For OSN, Collaboration, and PPI data we included all the nodes in selected clusters detected by modularity optimization and all edges between those nodes. In our experiments, these clusters are also used as input to compute expected modularity.

1. **Enron:** this dataset consists of emails sent between employees of Enron between 1999 and 2001. Nodes represent employees and there is an edge between two nodes if at least one email has been exchanged between them. The original dataset with edge probabilities is provided by the authors^7^.
2. **Protein-Protein Interaction (PPI):** the dataset contains nodes that represent proteins, edges that represent the interactions between two proteins, and associated probabilities. This dataset is extracted from a protein database that directly provides interaction probabilities^1^.
3. **Online Social Network (OSN):** the dataset is obtained from a weighted Facebook-like social network, that originates from an online community for students at the University of California, Irvine. The data, downloaded from toreopsahl.com, includes the users who sent or received at least one message. Probabilities are computed applying an exponential cumulative distribution function (CDF) of mean 2 to the weights^35^. We sample nodes and edges from the original networks.
4. **Collaboration:** the dataset is obtained from a weighted network of coauthorships between scientists posting preprints of the astrophysics archive at www.arxiv.org from 1995 to 1999^36^. We computed the probabilities using the same method as for the OSN data.

**Table 2:** Summary of real datasets.

Name	Nodes	Edges	Clusters
Enron	524	833	3
PPI	593	1185	5
OSN	306	1217	4
Collaboration	523	1224	2

### Accuracy and execution time

To verify the accuracy of our method, we compare the values of expected modularity computed using $\textrm{PWP}$ and $\textrm{FPWP}$ with the correct results, that we obtain using a brute-force approach iterating through all the possible worlds. It is worth noting that both $\textrm{PWP}$ and $\textrm{FPWP}$ are exact algorithms, that is, they are expected to compute the same result as the brute-force method (modulo numerical approximation): the experimental results are provided to show the correctness of our mathematical derivations.

For the first experiment, we generate networks with three communities and varying sizes, using a stochastic block model (also known as planted-community structure network)^37^, with the parameters shown in Table 3. We adjust $p_{in}$ and $p_{out}$ to control the size of the networks. For the second experiment, we generate random probabilistic networks, using Erdős-Rényi model, with different numbers of edges and we fix the number of communities to 5. Table 4 shows that the results calculated by $\textrm{PWP}$ and $\textrm{FPWP}$ are always the same as the true values, calculated by the brute-force method, down to several significant digits (four in the table). We also observe that even for larger networks where we can no longer use the brute-force method, the result obtained by $\textrm{FPWP}$ is the same as the one produced by $\textrm{PWP}$, as expected.

**Table 3:** Experimental parameters.

*m*	*n*	*k*	$\textrm{nc}$	$p_{in}$	$p_{out}$
9	9	3	3	0.8	0.03
14	12	3	4	0.8	0.03
21	15	3	5	0.6	0.04
25	18	3	6	0.5	0.03
35	21	3	7	0.5	0.03

*m*: number of edges; *n*: number of nodes; *k*: number of communities; $\textrm{nc}$: number of nodes in each community; $p_{in}$: edge density within communities; $p_{out}$: edge density between communities.

**Table 4:** Expected modularity computed using the brute-force method, $\textrm{PWP}$, and $\textrm{FPWP}$.

*m*	$Q^{\textrm{PWP}}$	$Q^{\textrm{FPWP}}$	$Q^{\text{BF}}$
9	0.3900	0.3900	0.3900
14	0.4401	0.4401	0.4401
21	0.4257	0.4257	0.4257
25	0.4455	0.4455	0.4455
35	0.4720	0.4720	–

*m*: number of edges; $Q^{\textrm{PWP}}$: expected modularity score computed by $\textrm{PWP}$; $Q^{\textrm{FPWP}}$: expected modularity score computed by $\textrm{FPWP}$; $Q^{BF}$: expected modularity score computed by the brute-force method.

**Fig. 5:**
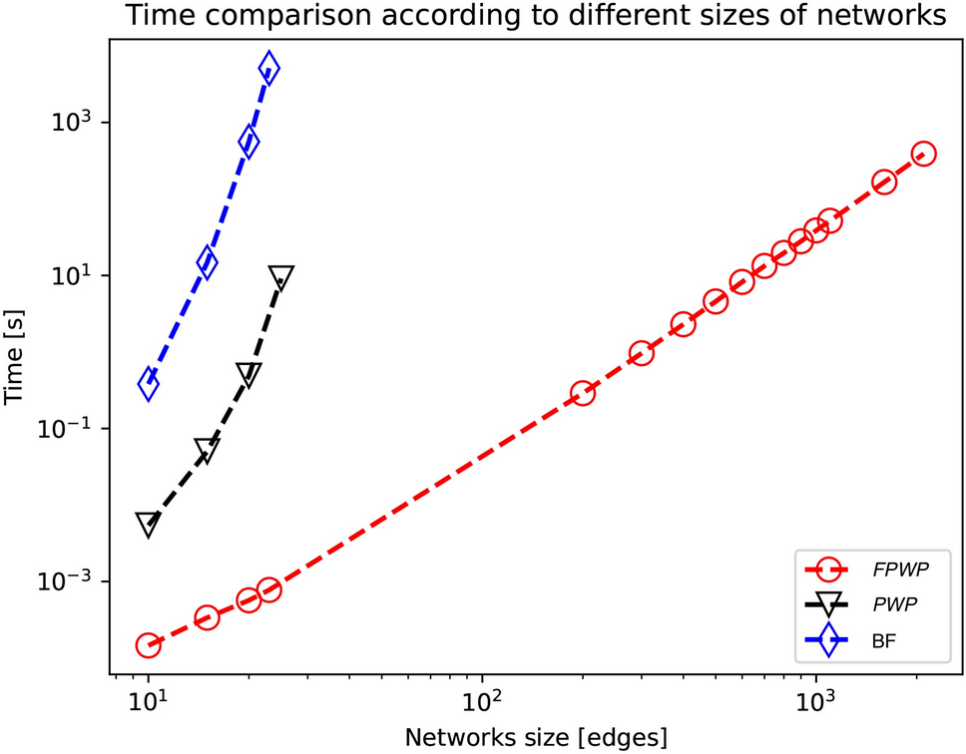
Running time of brute-force, $\textrm{PWP}$, and $\textrm{FPWP}$, with log axes.

Table 5 and Fig. 5 show that both our methods are multiple orders of magnitude faster than the brute-force approach. Figure 5 adds larger networks to test the behavior of $\textrm{FPWP}$ where the two other methods cannot be used. Both $\textrm{PWP}$ and the brute-force method have an exponential time complexity, although, $\textrm{PWP}$ is significantly faster. Figure 5 confirms the polynomial time complexity of $\textrm{FPWP}$.

**Table 5:** Running time of brute-force, $\textrm{PWP}$, and $\textrm{FPWP}$.

*m*	$T^{\text{PWP}}$	$T^{\text{FPWP}}$	$T^{\text{BF}}$
9	0.0020	0.0001	0.1201
14	0.0082	0.0004	8.7528
21	0.1916	0.0004	2401.2359
25	3.0294	0.0006	59956.8956
35	226.5636	0.0030	–

*m* = number of edges; $T^{\text{PWP}}$ = running time of $\textrm{PWP}$; $T^{\text{FPWP}}$ = running time of $\textrm{FPWP}$; $T^{\text{BF}}$ = running time of brute-force method

In the next sections we only use $\textrm{FPWP}$, given that it is both accurate and much faster than the brute-force method and $\textrm{PWP}$.

### Comparison with alternative methods

In this section we show that both weighting (that is, treating the probabilities as weights) and thresholding (that is, considering the network as a deterministic one after keeping only high-probability edges) lead to wrong estimations of expected modularity. On the contrary, sampling can be used to get more and more accurate results by increasing execution time, so we also study how the balance between time and accuracy when using a sampling approach compares to our algorithm.

#### Weighting

**Fig. 6:**
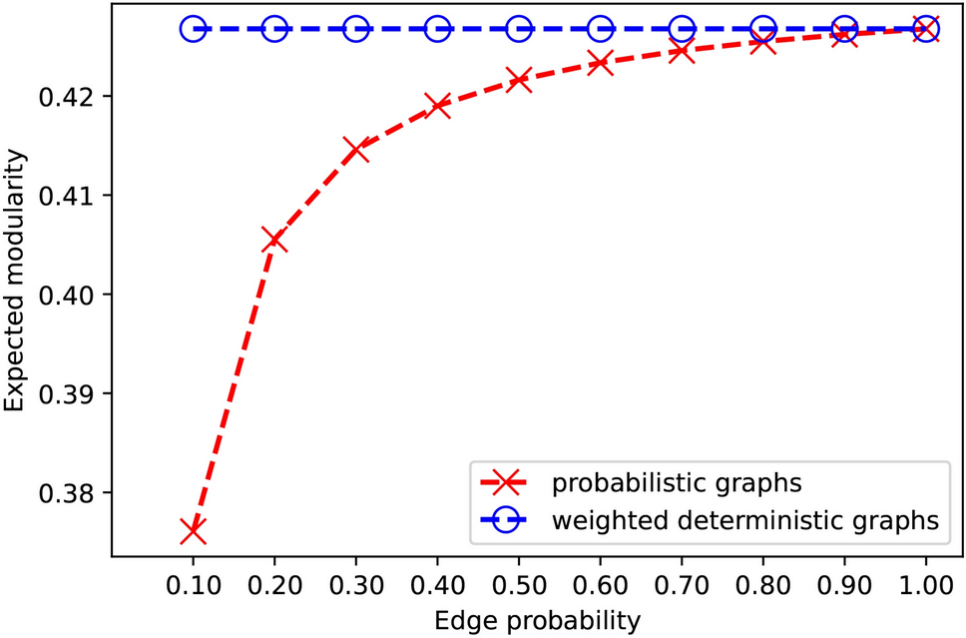
Weighted and expected modularity calculated on the same networks.

In this experiment, we show that directly regarding probabilistic networks as weighted deterministic networks leads to a wrong expected modularity calculation. In particular, we first generate a base network using stochastic block modeling, where the parameters are $k=3$, $\textrm{nc}=9$, $p_{in}=0.72$ and $p_{out}=0.12$. From this base network, we generate multiple probabilistic networks by assigning probabilities to its edges. In each probabilistic network we assign the same probability to all the edges: for example, in Fig. 6, the network corresponding to the value 0.20 on the *x* axis has all its edges having probability 0.20.

Figure 6 shows expected modularity computed using our algorithm (red) and the result obtained using weighted modularity. We can see that if we directly regard probabilistic networks as weighted deterministic networks, the final results are always the same for the different tested networks, and also different from expected modularity except for the case when the network is deterministic (where all edge probabilities equal 1).

#### Thresholding

In this experiment, we compare expected modularity (as computed by $\textrm{FPWP}$) with the modularity obtained after thresholding, showing that the choice of threshold largely influences the final results, which are also not accurate in general.

**Fig. 7:**
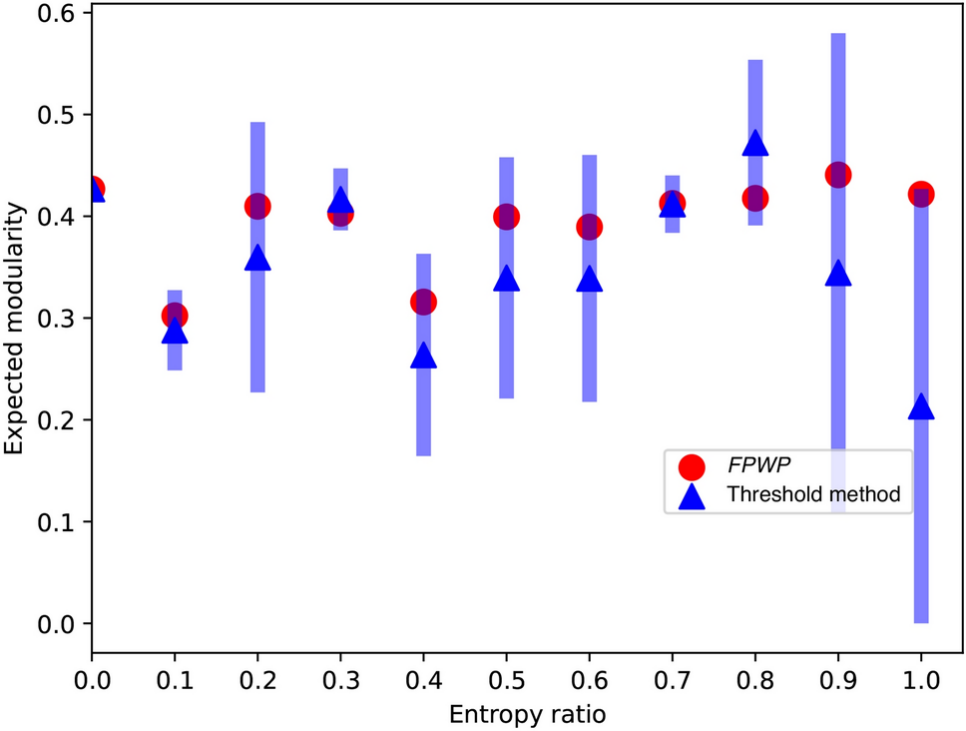
Mean and expected modularity calculated by $\textrm{FPWP}$ and using the threshold method.

**Fig. 8:**
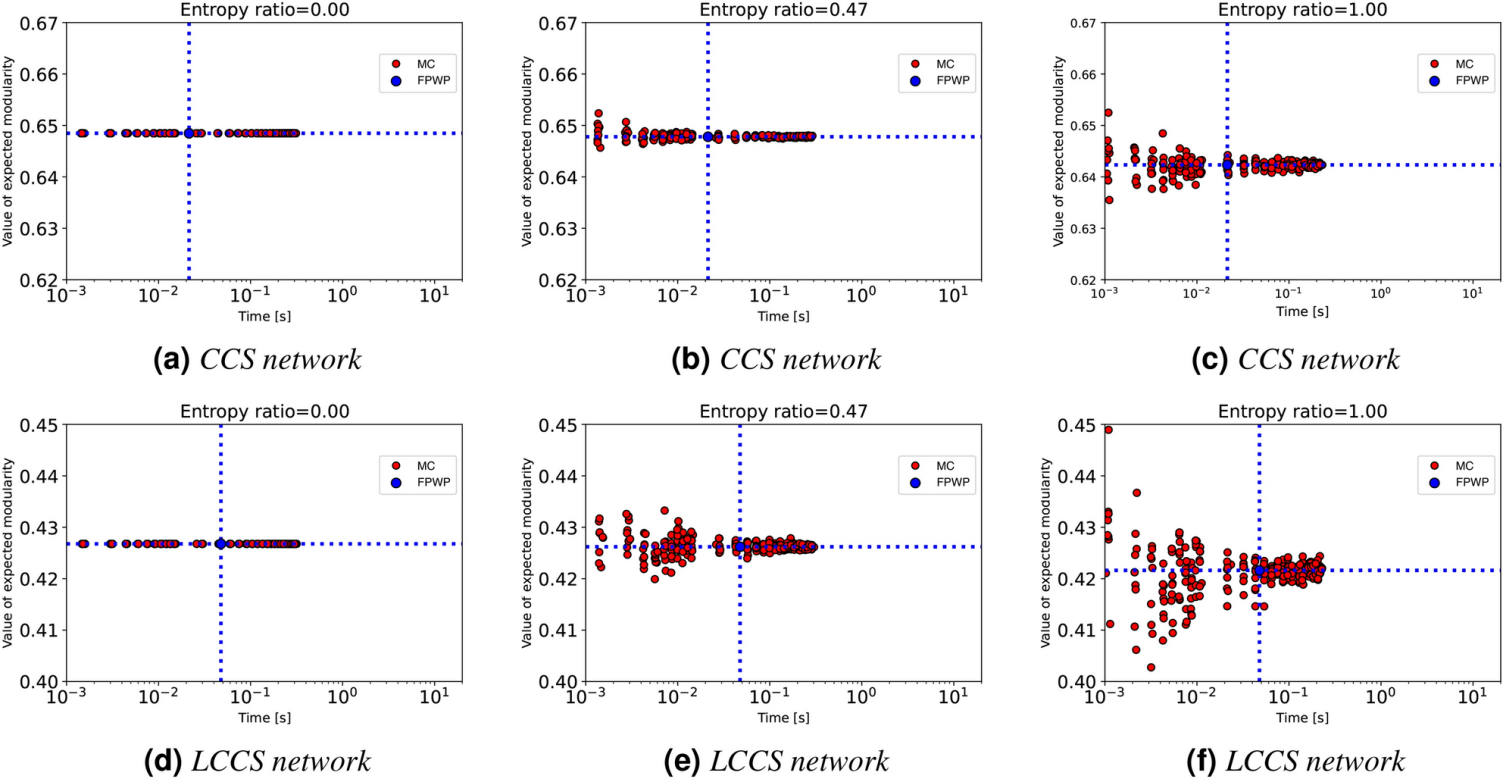
Running time and expected modularity value calculated using sampling and $\textrm{FPWP}$ for networks with a more clear (first row) and less clear (second row) modular structure, with different entropies. (**a**) CCS network with entropy ratio 0.00; (**b**) CCS network with entropy ratio 0.47; (**c**) CCS network with entropy ratio 1.00; (**d**) LCCS network with entropy ratio 0.00; (**e**) LCCS network with entropy ratio 0.47; (**f**) LCCS network with entropy ratio 1.00.

Here we generate planted community structure networks with 110 edges and 3 communities. All networks have the same topology, generated by a stochastic block model with $p_{in}=0.72$ and $p_{out}=0.12$. We then generate different probabilistic networks by assigning random probabilities to the edges so that, for different networks, entropy ratio ranges from 0 to 1, and randomly assign those probabilities to the edges. For each network, we compute the modularities obtained using different thresholds (from 0.1 to 1). In Fig. 7 we plot their mean (blue circles) and standard deviation (thick vertical lines). We can see that except for networks with low entropy ratios (that is, networks very close to deterministic networks), the modularities obtained through thresholding are far from the correct values (red line, calculated using $\textrm{FPWP}$).

#### Sampling

Thanks to the fact that using $\textrm{FPWP}$ we can compute expected modularity in polynomial time for a given input network, we can now test the ability of the sampling method to accurately estimate expected modularity, and study the factors influencing the balance between accuracy and execution time when sampling is used.

We use two kinds of random networks. One is characterized by a pronounced community structure, featuring dense intra-community edges and sparse inter-community edges (*CCS network*). The other exhibits a less distinct community structure, with less intra-community edges and more inter-community edges (*LCCS network*). Both networks contain 27 nodes and 3 communities. The *CCS network* and *LCCS network* are constructed with stochastic block models where the parameters are, respectively, $k=3$, $\textrm{nc}=9$, $p_{in}=0.99$, and $p_{out}=0.01$, and $k=3$, $\textrm{nc}=9$, $p_{in}=0.72$ and $p_{out}=0.12$. Notice that the CSS network closely approximates three cliques. When the entropy ratio equals 1.00, all edge probabilities are set to 0.50; when the entropy ratio equals 0.47, all edge probabilities are set to 0.90; when the entropy ratio is 0.00, all edges are deterministic.

Figure 8 shows the execution time (vertical blue line) and the computed expected modularity (blue horizontal line) for our algorithm. Each red circle is the result of a different execution of the sampling method. Specifically, the red nodes arranged in ‘bands’ are the result of different executions of the sampling method, all employing the same input $\theta$ as defined in Algorithm 1. For different executions, we have let the sampling method run for specific amounts of time (starting from the time needed by our algorithm), so that we could inspect the balance between execution time and likelihood to return an accurate result for the sampling method. For example, in Fig. 8f we can see eight executions of the sampling method close to the blue vertical line: these were stopped after the same time used by our algorithm to return, and their *y* value is the expected modularity they returned. From the figure we can see that, for different edge probability entropies, sampling always converges to the result produced by $\textrm{FPWP}$. Except for the case with no uncertainty (entropy ratio=0), sampling takes longer than $\textrm{FPWP}$ to converge toward the right results. By looking at Fig. 8a–c or Fig. 8d–f, we find that when entropy ratio increases (larger uncertainty), sampling converges more slowly. If we compare two figures on the same column, e.g. Fig. 8b, we find that when the community structure is less clear, the convergence time of sampling is longer.

### Factors influencing execution time of $\textrm{FPWP}$

**Fig. 9:**
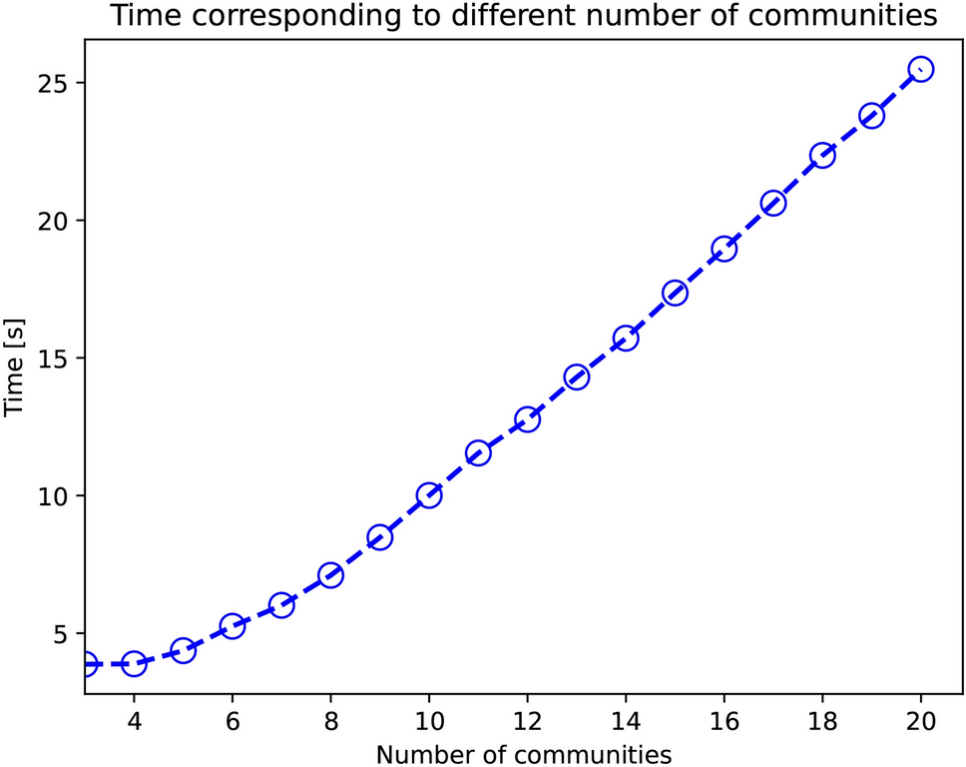
Time calculated by $\textrm{FPWP}$ with different numbers of communities.

In Fig. 9, we plot the execution time of $\textrm{FPWP}$ depending on the number of communities. For this experiment we generated a random probabilistic network with 100 nodes and 500 edges. We vary the number of communities from 3 to 20, randomly assigning the nodes to them. From this figure, we observe that as the number of communities increases, the execution time of $\textrm{FPWP}$ also increases.

**Fig. 10:**
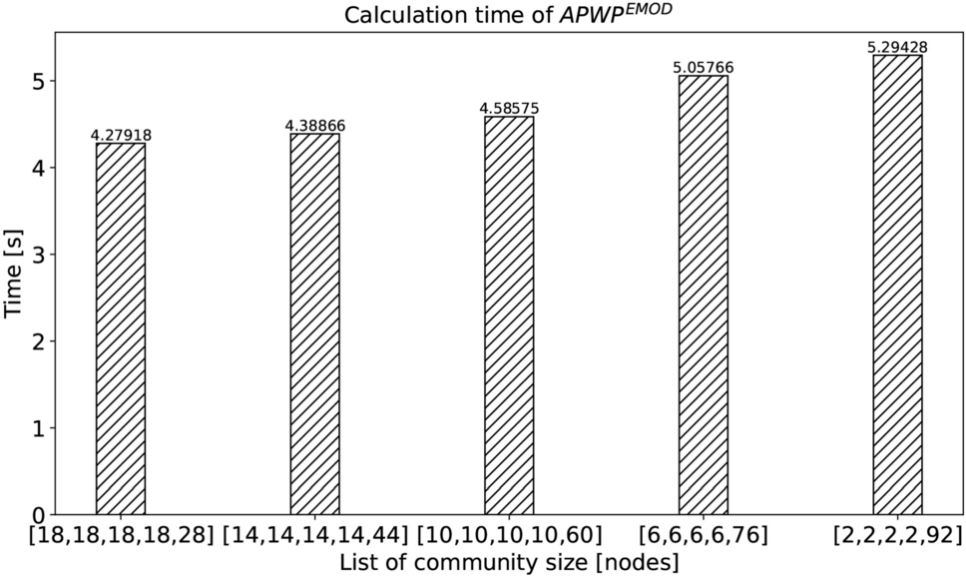
Time calculated by $\textrm{FPWP}$ with different variances of communities size.

In Fig. 10, we show the performance of $\textrm{FPWP}$ with the same number of communities but different community size distributions. From this figure, we can find that the larger variance among communities, the longer the running time.

In our final experiment, we examine the execution time of $\textrm{FPWP}$ on different random networks. In particular, we use 5 random network models: a Forest Fire Network (FFN)^38^, Barabási-Abert (BA)^39^, Small world (SW)^40^, Erdős-Rényi (ER)^41^, and a clear community-structure network (*CCS network*). We use each of these models to generate three random networks, all with around 200 nodes and 600 edges, but different numbers of communities. To provide a community assignment for the computation of modularity, we use a modularity optimization on the original deterministic networks. We also control the number of communities, where $|C|=4$, $|C|=5$ and $|C|=6$ respectively.

**Fig. 11:**
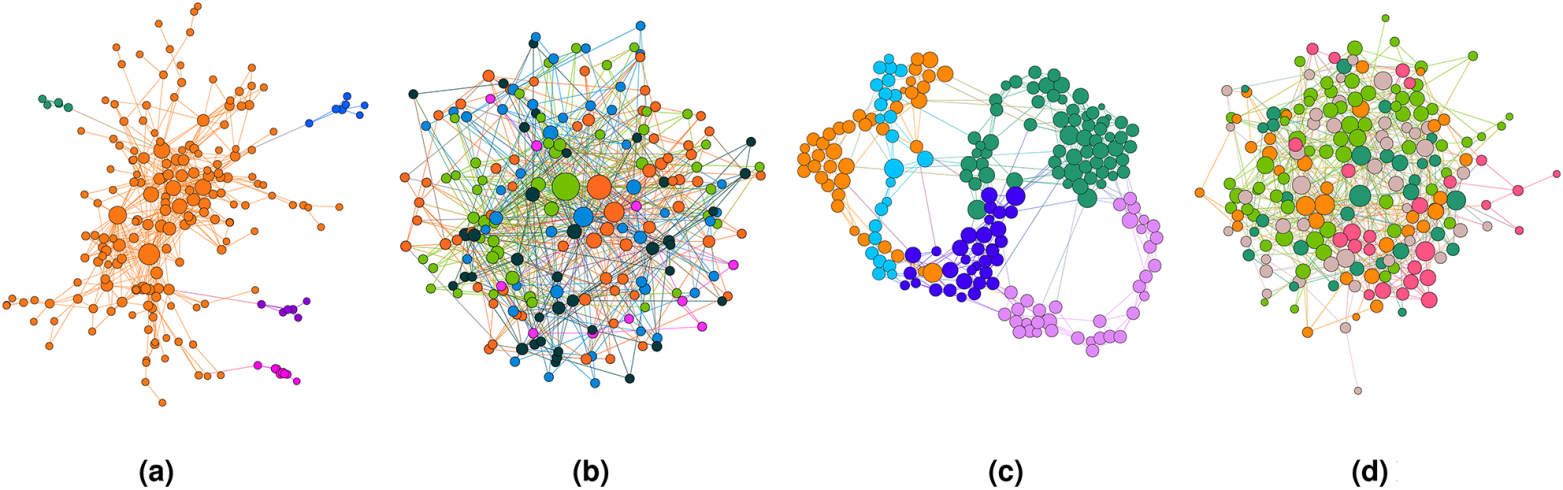
Networks generated by different models with 5 communities. (**a**) Forest Fire network; (**b**) Barabási-Abert network; (**c**) Small world network; and (**d**) Erdős-Rényi network.

Figure 11 shows different network structures containing 5 communities. Here nodes of the same color are in the same community computed using a modularity maximization method, and the number of colors represents the number of communities. Based on different network structures, we assign random edge probabilities so that entropy ratio is 0.4.

**Fig. 12:**
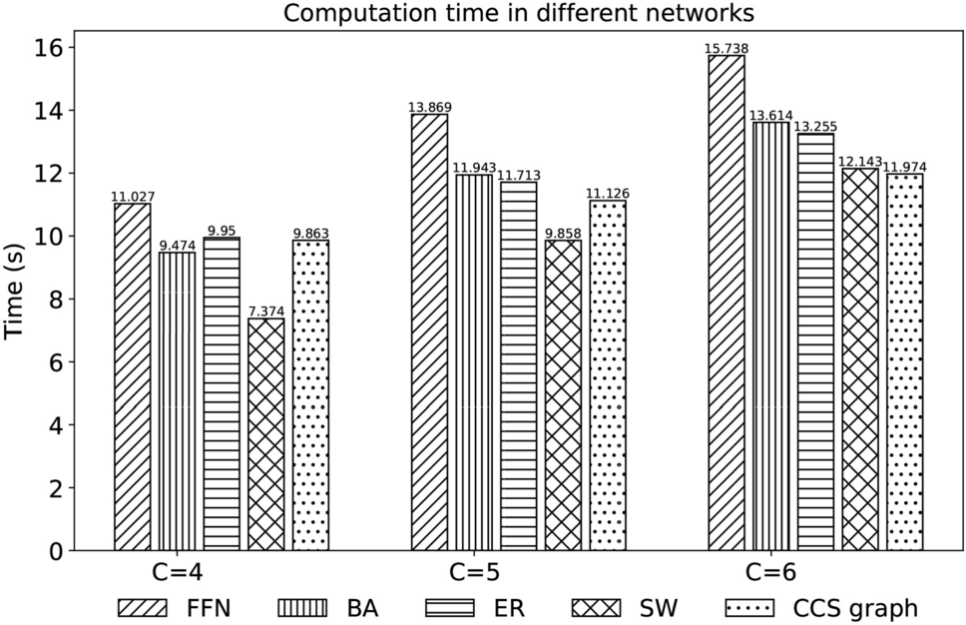
Running time of our method for different models.

Figure 12 shows the running time of $\textrm{FPWP}$ on networks with different network structures and numbers of communities. Generally, with an increasing number of communities the running time also increases, which fits the results in Fig. 9. The visualization in Fig. 11 suggests that a modularity optimization method may find a larger central community in FFN, which explains why the running time of FFN is always longer than for other networks. In fact, as shown in Fig. 10, the larger the community size variance, the longer the running time.

### Real data

**Fig. 13:**
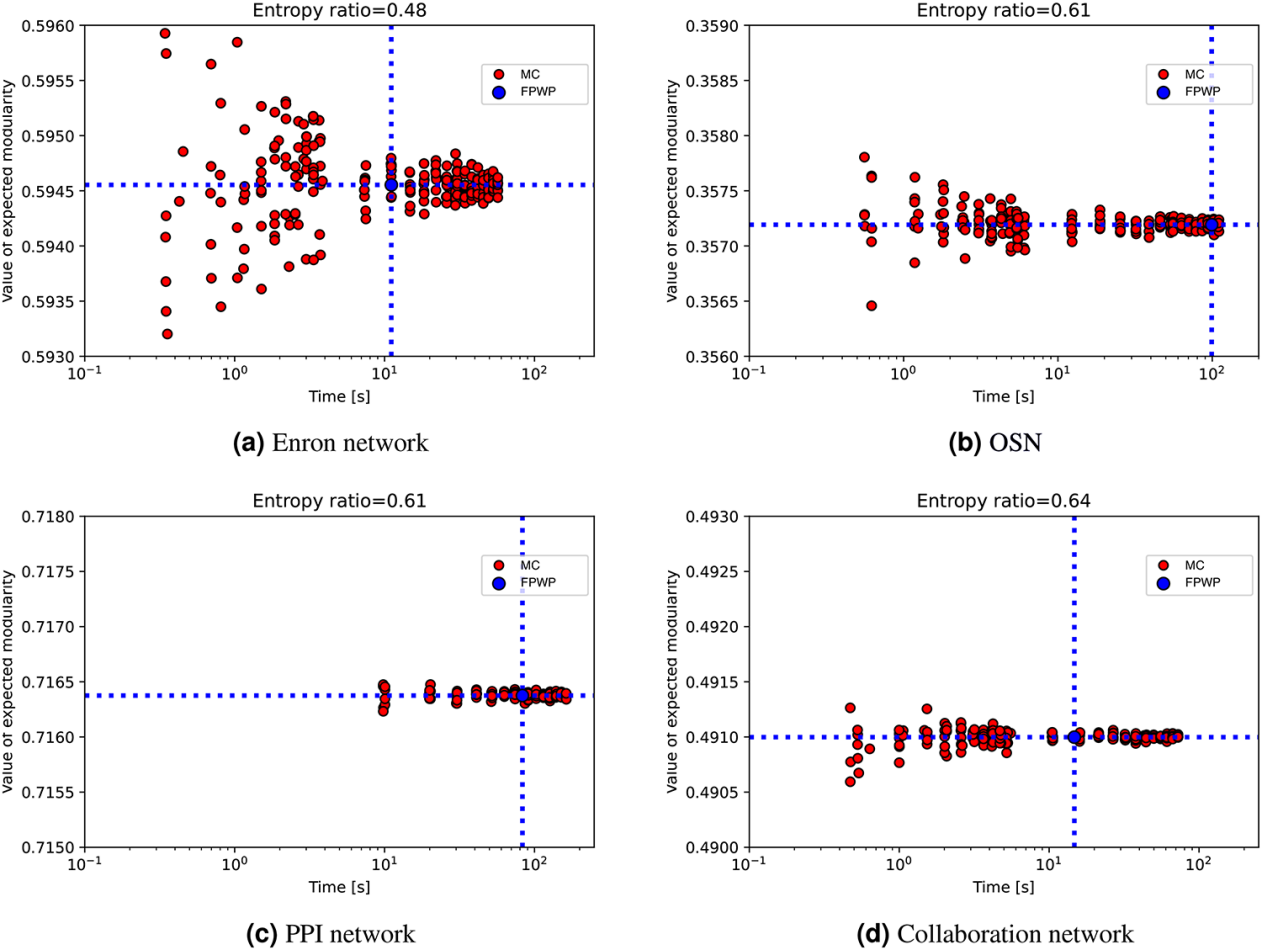
Running time and expected modularity value calculated by different methods in real datasets. (**a**) Enron network with entropy ratio 0.48; (**b**) OSN network with entropy ratio 0.61; (**c**) PPI network with entropy ratio 0.61; (**d**) Collaboration network with entropy ratio 0.64.

**Fig. 14:**
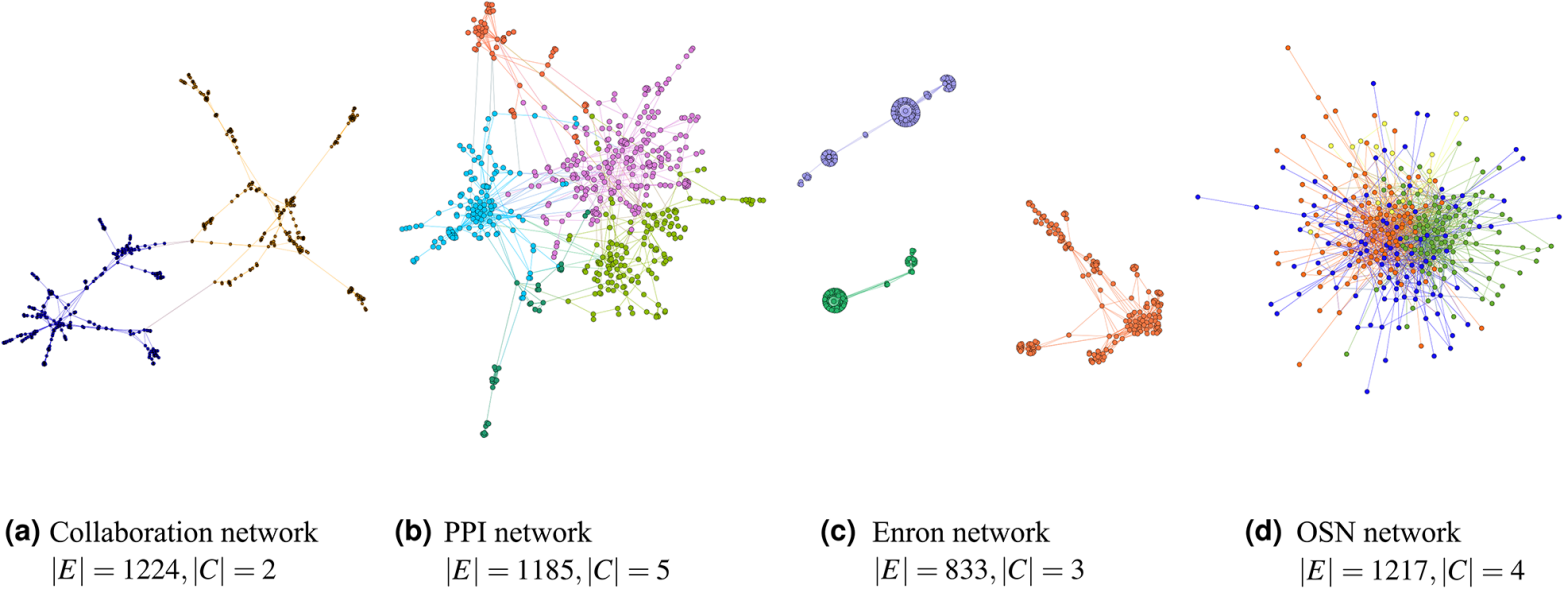
Real networks and the communities used in the experiments. (**a**) Collaboration network; (**b**) PPI network; (**c**) Enron network; (**d**) OSN network.

In this section, we apply $\textrm{FPWP}$ and the sampling method on real networks. Notice that we do not use thresholding and weighting, because the experiments on synthetic data have clearly shown that these approaches should not be used to compute expected modularity. Figure 14 shows different network structures with different numbers of clusters. Here nodes of the same color are in the same community computed using a modularity maximization method, and the number of colors represents the number of communities. Figure 13 shows the execution time (vertical blue line) and the computed expected modularity (blue horizontal line) for our algorithm. Each red circle is the result of a different execution of the sampling method. For different executions, we have let the sampling method run for different amounts of time, so that we could inspect the balance between execution time and the likelihood of returning an accurate result for the sampling method. Figure 13 shows the same trends seen in the experiments with synthetic data. In these datasets, where the probability distributions are fixed and where clear community structures exist (we remind the reader that real data was obtained from high-modularity networks), the sampling method can produce accurate results in a short time, in accordance with our results on synthetic data.

## Discussion

In this paper, we evaluate alternative approaches for the calculation of expected modularity in probabilistic networks, including an original approach compatible with possible worlds semantics.

When compared with a brute-force approach, our new approach is several orders of magnitude faster, with $\textrm{FPWP}$ reducing time complexity from exponential to polynomial without affecting accuracy. For a network with 25 edges, which is around the limit of what we could handle using exact computation methods before this paper, the execution time goes down from more than 15 h (brute-force) to less than 5 s ($\textrm{PWP}$) to a few microseconds ($\textrm{FPWP}$). The execution time is however still high for large networks. In cases when the computation has to be repeated multiple times sequentially, for example inside unoptimized expected modularity maximization algorithms, one may have to consider using faster but less accurate approaches.

We show that considering probabilities as weights, which can be a useful approach for other tasks, should not be used to compute expected modularity because it returns wrong results in general. The main reason is that node strengths (which equal node expected degrees in probabilistic networks) are multiplied in weighted modularity computation, while the expected product of degrees is not the same as the product of expected degrees in case of dependencies. Unfortunately, degrees in probabilistic networks are not independent, even when edge probabilities are, because edges are incident to two nodes and thus they either contribute to the degree of both or of none of them. A second reason is that weights are interpreted relative to each other in weighted modularity. So, for example, having all weights set to 0.01 or having them set to 1 makes no difference, while interpreting these numbers as probabilities means that in one case the network is probably empty, while in the second case all edges are almost certainly there. Identifying cases where using weighted modularity instead of expected modularity can still be appropriate is an interesting research question raised by this work.

Thresholding is a common and very fast approach to handle probabilistic networks. In fact, we argue that even if the large majority of network studies currently use deterministic networks, these are in fact often uncertain networks where some more or less explicit decisions have been made about including or not edges based on the available information about the modeled system—that is, some thresholding has been performed. In this paper we show that thresholding is not a good approach for the task of computing expected modularity in general. However this is the case only when the network is almost deterministic. If we assume that all probabilities are at distance $\epsilon$ from 0 or 1, using thresholding we change edge probabilities from $1 - \epsilon$ to 1 and from $\epsilon$ to 0, for all thresholds between $\epsilon$ and $1 - \epsilon$. With small values of $\epsilon$, the change is small and the range of good thresholds is wide, making the method fast, accurate, and robust. However, as we are interested in handling probabilistic networks, it is not very useful to have a method that only works when the network is practically not probabilistic.

While the amount of uncertainty has practically no effect on the accuracy and execution time of the new method we introduce in this paper, this is an important factor when sampling is used. This feature of sampling is already well acknowledged in the literature, where some methods have been proposed to tranform the original network into another probabilistic network of lower entropy^29,42^. Sampling from a probabilistic network with probabilities very close to 0 or 1 immediately gives an accurate result, the same computed on the thresholded deterministic network. We have also noticed how the likelihood of producing an accurate value of expected modularity using sampling increases when the community structure is very clear, close to a set of cliques. This is due to the fact that modularity depends on the number of edges inside communities, and not on the position of these edges. As a result, when many (and most) edges are inside communities, many sampled networks will have a similar number of edges inside those communities, and thus similar values of modularity. Sampling from a narrow distribution has then a faster convergence. However, sampling can produce an inaccurate result, in addition to not giving any indication of whether the result is accurate or not. The more the network is uncertain, the less well sampling works.

While the probability distribution does not impact the execution time of our method, the way in which nodes are assigned to communities does, both with respect to the number of communities and to the distribution of community sizes. The experiments on different types of networks (with or without a long-tail degree distribution, with or without a high clustering coefficient) also show that our algorithms are not significantly and directly affected by the network type, although depending on the network type it can be more or less likely to have to execute our algorithm with large communities, which would then indirectly affect execution time.

This paper opens the problem of how to use methods for the computation of expected modularity as a sub-procedure of a community detection method. Such an algorithm has not been proposed yet, despite the popularity of its deterministic counterpart. So far, clustering algorithms for probabilistic networks have been mostly developed outside of the network science research community, overlooking the popularity of the modularity objective function^12,16,43,44^. This problem adds two interesting aspects to our work: first, when used as a heuristic inside an optimization algorithm, a less accurate calculation of modularity may still lead to the same clustering, so there is a question about when it is needed to be accurate and when a faster approximation is sufficient. That is, both sampling and considering probabilities as weights could be usable inside a community detection algorithm, even if their results may be inaccurate. Second, we may have to execute the modularity calculation many times, which poses an additional computational challenge in particular for large networks. These two aspects may allow the usage of different computational approaches^45,46^.

## Data Availability

The datasets generated and analysed during the current study are available in the repository: https://github.com/XINS3/Expected-modularity-calculation-over-uncertain-graph.

## Code availability

The code to replicate the experiments is available here: https://github.com/XINS3/Expected-modularity-calculation-over-uncertain-graph.

## References

[1] Krogan, N. J. et al. Global landscape of protein complexes in the yeast saccharomyces cerevisiae. *Nature* **440**, 637–643 (2006). 16554755 10.1038/nature04670

[2] Danesh, M., Dorrigiv, M. & Yaghmaee, F. DGCU: A new deep directed method based on gaussian embedding for clustering uncertain graphs. *Comput. Electr. Eng.* **101**, 108066 (2022).

[3] Yu, D. et al. Stable structural clustering in uncertain graphs. *Inf. Sci.* **586**, 596–610 (2022).

[4] Farine, D. R. & Strandburg-Peshkin, A. Estimating uncertainty and reliability of social network data using Bayesian inference. *R. Soc. Open Sci.* **2**, 150367 (2015). 26473059 10.1098/rsos.150367 PMC4593693

[5] Liu, L., Jin, R., Aggarwal, C., Shen, Y. Reliable clustering on uncertain graphs. In *2012 IEEE 12th International Conference on Data Mining*, 459–468 (IEEE, 2012).

[6] Frank, H. Shortest paths in probabilistic graphs. *Oper. Res.* **17**, 583–599 (1969).

[7] Kaveh, A., Magnani, M. & Rohner, C. Defining and measuring probabilistic ego networks. *Soc. Netw. Anal. Min.* **11**, 1–12 (2021).

[8] Ceccarello, M., Fantozzi, C., Pietracaprina, A., Pucci, G. & Vandin, F. Clustering uncertain graphs. *Proc. VLDB Endow.* **11**, 472–484 (2017).

[9] Danesh, M., Dorrigiv, M. & Yaghmaee, F. A survey of clustering large probabilistic graphs: Techniques, evaluations, and applications. *Expert Syst.* **40**, e13248 (2023).

[10] Hussain, S. F. & Maab, I. Clustering probabilistic graphs using neighbourhood paths. *Inf. Sci.* **568**, 216–238 (2021).

[11] Hussain, S. F., Butt, I. A., Hanif, M. & Anwar, S. Clustering uncertain graphs using ant colony optimization (ACO). *Neural Comput. Appl.* **34**, 11721–11738 (2022).

[12] Liang, Y., Hu, T., Zhao, P. Efficient Structural clustering in large uncertain graphs. In *2020 IEEE 36th International Conference on Data Engineering (ICDE)*, 1966–1969. 10.1109/ICDE48307.2020.00215 (2020).

[13] Danesh, M., Dorrigiv, M. & Yaghmaee, F. Ensemble-based clustering of large probabilistic graphs using neighborhood and distance metric learning. *J. Supercomput.* **77**, 4107–4134 (2021).

[14] Pileggi, S.F. A cross-domain perspective to clustering with uncertainty. In *International Conference on Computational Science*, 295–308 (Springer, 2024).

[15] Banerjee, S. A survey on mining and analysis of uncertain graphs. *Knowl. Inf. Syst.* **64**, 1653–1689 (2022).

[16] Kollios, G., Potamias, M. & Terzi, E. Clustering large probabilistic graphs. *IEEE Trans. Knowl. Data Eng.* **25**, 325–336 (2011).

[17] Halim, Z., Waqas, M. & Hussain, S. F. Clustering large probabilistic graphs using multi-population evolutionary algorithm. *Inf. Sci.* **317**, 78–95 (2015).

[18] Halim, Z. & Khattak, J. H. Density-based clustering of big probabilistic graphs. *Evol. Syst.* **10**, 333–350 (2019).

[19] Newman, M. E. Modularity and community structure in networks. *Proc. Natl. Acad Sci.* **103**, 8577–8582 (2006). 16723398 10.1073/pnas.0601602103 PMC1482622

[20] Guimera, R., Sales-Pardo, M. & Amaral, L. A. N. Modularity from fluctuations in random graphs and complex networks. *Phys. Rev. E* **70**, 025101 (2004). 10.1103/PhysRevE.70.025101 PMC2441765 15447530

[21] McDiarmid, C. & Skerman, F. Modularity of Erdős-Rényi random graphs. *Random Struct. Algorithms* **57**, 211–243 (2020).

[22] Hanteer, O., & Magnani, M. Unspoken assumptions in multi-layer modularity maximization. *Sci. Rep.* **10**, 11053. 10.1038/s41598-020-66956-0 (2020). Number: 1 Publisher: Nature Publishing Group. 10.1038/s41598-020-66956-0 PMC7338500 32632217

[23] Peixoto, T. P. *Descriptive Versus Inferential Community Detection in Networks: Pitfalls, Myths and Half-Truths* (Cambridge University Press, Cambridge, 2023).

[24] Chen, M., Kuzmin, K. & Szymanski, B. K. Community detection via maximization of modularity and its variants. *IEEE Trans. Comput. Soc. Syst.* **1**, 46–65 (2014).

[25] Abiteboul, S., Kanellakis, P. & Grahne, G. On the representation and querying of sets of possible worlds. *Theor. Comput. Sci.* **78**, 159–187 (1991).

[26] Potamias, M., Bonchi, F., Gionis, A. & Kollios, G. K-nearest neighbors in uncertain graphs. *Proc. VLDB Endow.* **3**, 997–1008 (2010).

[27] Boonma, P., Natwichai, J. Reliable cluster on uncertain multigraph. In *2015 18th International Conference on Network-Based Information Systems*, 494–498 (IEEE, 2015).

[28] Parchas, P., Gullo, F., Papadias, D. & Bonchi, F. Uncertain graph processing through representative instances. *ACM Trans. Database Syst. (TODS)* **40**, 1–39 (2015).

[29] Parchas, P., Papailiou, N., Papadias, D. & Bonchi, F. Uncertain graph sparsification. *IEEE Trans. Knowl. Data Eng.* **30**, 2435–2449 (2018).

[30] Kaveh, A. *Modelling and Analysis of Probabilistic Networks*. Ph.D. thesis, Acta Universitatis Upsaliensis (2022).

[31] Ghosh, J., Ngo, H.Q., Yoon, S., & Qiao, C. On a routing problem within probabilistic graphs and its application to intermittently connected networks. In *IEEE INFOCOM 2007-26th IEEE International Conference on Computer Communications*, 1721–1729 (IEEE, 2007).

[32] Wing, O. & Demetriou, P. Analysis of probabilistic networks. *IEEE Trans. Commun. Technol.* **12**, 38–40 (1964).

[33] Li, R.-H., Yu, J.X., Mao, R., & Jin, T. Recursive Stratified Sampling: A New Framework for Query Evaluation on Uncertain Graphs. *IEEE Trans. Knowl. Data Eng.* **28**, 468–482. 10.1109/TKDE.2015.2485212 (2016). Publisher: IEEE Computer Society.

[34] Fernández, M. & Williams, S. Closed-form expression for the Poisson-binomial probability density function. *IEEE Trans. Aerospace Electron. Syst.* **46**, 803–817 (2010).

[35] Li, R.-H., Yu, J. X., Mao, R. & Jin, T. Recursive stratified sampling: A new framework for query evaluation on uncertain graphs. *IEEE Trans. Knowl. Data Eng.* **28**, 468–482 (2015).

[36] Newman, M. E. The structure of scientific collaboration networks. *Proc. Natl. Acad Sci.* **98**, 404–409 (2001). 11149952 10.1073/pnas.021544898 PMC14598

[37] Condon, A. & Karp, R. M. Algorithms for graph partitioning on the planted partition model. *Random Struct. Algorithms* **18**, 116–140 (2001).

[38] Leskovec, J., Kleinberg, J., & Faloutsos, C. Graphs over time: densification laws, shrinking diameters and possible explanations. In *Proceedings of the Eleventh ACM SIGKDD International Conference on Knowledge Discovery in Data Mining*, 177–187 (2005).

[39] Barabási, A.-L. & Albert, R. Emergence of scaling in random networks. *Science* **286**, 509–512 (1999). 10521342 10.1126/science.286.5439.509

[40] Watts, D. J. & Strogatz, S. H. Collective dynamics of ‘small-world’networks. *Nature* **393**, 440–442 (1998). 9623998 10.1038/30918

[41] Erdős, P. et al. On the evolution of random graphs. *Publ. Math. Inst. Hung. Acad. Sci* **5**, 17–60 (1960).

[42] Kaveh, A., Magnani, M. & Rohner, C. Probabilistic network sparsification with ego betweenness. *Appl. Netw. Sci.* **6**, 1–21. 10.1007/s41109-02100401-7 (2021).

[43] Qiu, Y.-X. *et al.* Efficient structural clustering on probabilistic graphs. *IEEE Trans. Knowl. Data Eng.* **31**, 1954–1968. 10.1109/TKDE.2018.2872553 (2019). Conference Name: IEEE Transactions on Knowledge and Data Engineering.

[44] Han, K. et al. Efficient and effective algorithms for clustering uncertain graphs. *Proc. VLDB Endow.* **12**, 667–680. 10.14778/3311880.3311884 (2019).

[45] Qiao, S. et al. A fast parallel community discovery model on complex networks through approximate optimization. *IEEE Trans. Knowl. Data Eng.* **30**, 1638–1651 (2018).

[46] Tian, F., Gao, B., Cui, Q., Chen, E., & Liu, T.-Y. Learning deep representations for graph clustering. In *Proceedings of the AAAI Conference on Artificial Intelligence* (2014).

